# The use of mixture density networks in the emulation of complex epidemiological individual-based models

**DOI:** 10.1371/journal.pcbi.1006869

**Published:** 2020-03-16

**Authors:** Christopher N. Davis, T. Deirdre Hollingsworth, Quentin Caudron, Michael A. Irvine

**Affiliations:** 1 MathSys CDT, Mathematics Institute, University of Warwick, Coventry, United Kingdom; 2 Zeeman Institute (SBIDER), University of Warwick, Coventry, United Kingdom; 3 Big Data Institute, Li Ka Shing Centre for Health Information and Discovery, Nuffield Department of Medicine, University of Oxford, Oxford, United Kingdom; 4 Scai Analytics Ltd., Vancouver, Canada; 5 Institute of Applied Mathematics, University of British Columbia, Vancouver, Canada; University of Florida, UNITED STATES

## Abstract

Complex, highly-computational, individual-based models are abundant in epidemiology. For epidemics such as macro-parasitic diseases, detailed modelling of human behaviour and pathogen life-cycle are required in order to produce accurate results. This can often lead to models that are computationally-expensive to analyse and perform model fitting, and often require many simulation runs in order to build up sufficient statistics. Emulation can provide a more computationally-efficient output of the individual-based model, by approximating it using a statistical model. Previous work has used Gaussian processes (GPs) in order to achieve this, but these can not deal with multi-modal, heavy-tailed, or discrete distributions. Here, we introduce the concept of a mixture density network (MDN) in its application in the emulation of epidemiological models. MDNs incorporate both a mixture model and a neural network to provide a flexible tool for emulating a variety of models and outputs. We develop an MDN emulation methodology and demonstrate its use on a number of simple models incorporating both normal, gamma and beta distribution outputs. We then explore its use on the stochastic SIR model to predict the final size distribution and infection dynamics. MDNs have the potential to faithfully reproduce multiple outputs of an individual-based model and allow for rapid analysis from a range of users. As such, an open-access library of the method has been released alongside this manuscript.

## Author summary

Infectious disease modellers have a growing need to expose their models to a variety of stakeholders in interactive, engaging ways that allow them to explore different scenarios. This approach can come with a considerable computational cost that motivates providing a simpler representation of the complex model. We propose the use of mixture density networks as a solution to this problem. MDNs are highly flexible, deep neural network-based models that can emulate a variety of data, including counts and over-dispersion. We explore their use firstly through emulating a negative binomial distribution, which arises in many places in ecology and parasite epidemiology. Then, we explore the approach using a stochastic SIR model. We also provide an accompanying Python library with code for all examples given in the manuscript. We believe that the use of emulation will provide a method to package an infectious disease model such that it can be disseminated to the widest audience possible.

This is a *PLOS Computational Biology* Methods paper.

## Introduction

Complex individual-based models abound in epidemiology. These can often include a mixture of different distributions leading to a high-dimensional output space with possibly multi-modal distributions. As an example, consider the relatively simple stochastic Susceptible-Infected-Recovered (SIR) model [1]. In the SIR model, individuals become infected stochastically with probability depending on the number of infected individuals in the population. Once infected, they contribute to further transmissions, until they eventually recover with immunity. For some parameter regimes, depending on initial conditions and stochastic variation, a large outbreak (epidemic) may be observed, or alternatively, the infected individuals may recover before a large-scale epidemic can occur (stochastic fade-out) [2]. Thus, we would see a multi-modal distribution of the cumulative number of infected individuals depending on whether the outbreak results in an epidemic or stochastic fade-out.

Other examples of complex, individual-based models occur in macro-parasitic diseases. These diseases will often have highly heterogeneous parasite distributions amongst its hosts, which therefore requires explicit individual hosts to be modelled in order to directly understand the consequences and implications of these distributions [3]. Compounding this, many of these diseases are controlled through mass drug administration, where coverage, adherence and demographic factors can play a role in the outcome of a program [4, 5].

Finally, there are many examples of individual-based models for sexually-transmitted infectious disease, such as HIV, HPV, gonorrhea, and syphilis [6]. Here, complexities such as heterogeneity in risk, partnership-formation, sexual contact networks, sero-sorting behaviour, and heterogeneity in interventions can lead to dramatically different outcomes that require modelling.

Coupled with the increasing number of computationally-expensive models is the move towards models that are more accessible, such that non-experts can explore the key concepts and outputs. There has been an increasing call for more models to be outward facing to be used by policy makers and other non-modellers [7]. Despite this, there remains significant technological barriers to be able to perform this in general, often requiring skill in multiple programming languages and software development [8]. In particular, one of the technological barriers is the speed at which model simulations of a given scenario can occur. Thus, this introduces the idea of using emulation in lieu of model computation [9–13], where we replace the individual-based model with a statistical model that is more computationally efficient to sample from. These statistical models can be difficult to train and often require the assumption that the model produces unimodal or normal outputs [14].

As individual-based models become more complex, the necessary computational costs increase. This can often lead to only a small number of scenarios being explored with relatively few replicates used to estimate uncertainty within the model. Inference schemes, like approximate Bayesian computation, where many simulation runs need to be performed, will have a particularly high computational cost [15]. Computational speed-up can be performed by making certain approximations within the model, such as taking the deterministic limit for a process that has relatively large numbers, or, again, through the use of emulation [4, 14].

The main concept of an emulator is to fit a statistical regression model to the inputs and outputs of the individual-based model and then evaluate the computationally much faster model instead [16]. For example, an individual-based model may have *m* number of inputs (*x*_0_, …, *x*_*m*−1_), with a corresponding single output *y*. If we assume that, for a given set of inputs, the output is normally-distributed, we can then emulate the model using the following linear regression

$$\begin{array}{c} y=\sum_{k=0}^{m-1}\beta_{k}x_{k}+\epsilon , \end{array}$$ (1)

where *ϵ* ∼ *N*(0, *σ*^2^). However, for all but simple individual-based models, the linearity assumption may be too restrictive. The fixed *β*_*k*_*x*_*k*_ terms may be replaced with a Gaussian process (GP), where values can vary across the input space, with the assumption that the closer input points are together, the more correlated will be the outputs. The use of GPs for emulation of epidemiological models has seen several successes [14, 16–19].

Yet, emulating with GPs has a number of disadvantages. Firstly, they assume that the outputs of a model for a given set of inputs are normal. Therefore, they cannot take into account the multi-modality or heavy-tailedness of certain data. There may also be some restrictive assumptions on the smoothness of the correlation between two points in input space. We propose the use of a mixture density network (MDN) to overcome some of these issues. We replace the linear regression component with a neural network that is flexible enough to capture complex relationships and replace the simple normal distribution with a mixture of distributions that provides a more general family of distributions for the model output [20].

The advantage of allowing the model output to be given with a complicated distributional form, however, does mean that a large data set is required to train the model. This may introduce difficulties in the use of MDNs for the most computationally expensive models, as many simulations will be needed to initially obtain this data set, particularly when the input and output dimensions are large and the training data needs to cover this vast space. We note carefully set up GP approaches have been used for emulating models with a high input dimension [21]. Furthermore, GPs can estimate the uncertainty of the emulator, which can then be incorporated in decision support calculations. On the other hand, MDNs do not do this directly and so only by using a data set large enough that the uncertainty introduced by the emulator is small compared to other uncertainties, can MDNs be safely used. A summary of the comparison between GPs and MDNs is shown in Table 1.

**Table 1:** A comparison of mixture density networks and Gaussian processes.

Mixture density network	Gaussian process
Can emulate multi-modal distributions.	Only suitable for uni-modal distributions.
Flexible output distribution mixture allows for application to different data types, such as overly-dispersed, finite domain or discrete.	Output distribution is normal.
Requires large training set to capture output.	Suitable for small data sets.
Good scaling properties.	Scales poorly with data size.
Hyperparameters need to be tuned in training process.	Hyperparameters need to be tuned in training process.
The training process is stochastic.	For given parameters, the method is optimised exactly.
The uncertainty is hard to quantify.	Directly measures uncertainty.

The manuscript is segmented as follows: we first introduce the concept of an MDN, and how it relates to an individual-based model, and apply the concept to a number of simple examples to demonstrate its use. We then consider a stochastic SIR model and emulate the final size distribution using an MDN. Finally, we demonstrate its use on estimating the distribution of susceptible and infected individuals in a model with vaccination. All analyses presented within the manuscript were conducted within the package framework and example code is given (see https://github.com/QCaudron/pydra).

## Methods

### Introduction to mixture density networks

Mixture density networks (MDNs) are built from two components: a neural network and a mixture model. We begin by introducing the concept of a mixture model—a model of probability distributions built up from a weighted sum of more simple distributions. More concretely, we consider a one-dimensional distribution with *m* mixture components. The probability density function (PDF) *p*(*x*) is represented by the *m* PDFs indexed by *j* *p*_*j*_(*x*), with weights Ω = {*ω*_0_, …, *ω*_*m*−1_}, where $\sum_{j=0}^{m-1}\omega_{j}=1$, by the following equation

$$\begin{array}{c} p\left(x\right)=\sum_{j=0}^{m-1}\omega_{j}p_{j}\left(x\right). \end{array}$$ (2)

Typically these probability distributions will be parameterised by a series of parameters that reflect the shape and location of the distribution Θ = {*θ*_0_, …, *θ*_*m*−1_}. The full parameterised model may therefore be written as

$$\begin{array}{c} p\left(x|\Omega ,\Theta \right)=\sum_{j=0}^{m-1}\omega_{j}p_{j}\left(x|\theta_{j}\right). \end{array}$$ (3)

As an example, each *p*_*j*_ could be a normal distribution parameterised by a mean *μ*_*j*_ and a variance *σ*_*j*_. The mixture model would then have the following form,

$$\begin{array}{c} p\left(x|\Omega ,\Theta \right)=\sum_{j=0}^{m-1}\frac{\omega_{j}}{\sqrt{2\pi \sigma_{j}^{2}}}\exp(\frac{-1}{2\sigma_{j}^{2}}{\left(x-\mu_{j}\right)}^{2}). \end{array}$$ (4)

In general, these mixture distributions are multi-modal, including those in the form of Eq (4), and can be fitted directly to some data **x** = (*x*_0_, …, *x*_*n*−1_). Assuming independence, the corresponding likelihood is calculated as

$$\begin{array}{c} l\left(x|\Omega ,\Theta \right)=\prod_{i=0}^{n-1}[\sum_{j=0}^{m-1}\omega_{j}p_{j}\left(x_{i}|\theta_{j}\right)]. \end{array}$$ (5)

Fitting can then typically proceed using expectation–maximisation [20]. For our purposes, we have an individual-based model *M*, with some input *α*, that produces stochastic realisations *y* ∼ *M*(*α*). We therefore wish to derive a relationship between the input parameters *α*, and the mixture density weights *ω*_*j*_(*α*) and density parameterisations *θ*_*j*_(*α*). This could potentially be done with a separate regression for each of the density parameters and weights, although this would fail to capture the corresponding relationships that would exist between each parameter and weight. We can therefore model these using a neural network, which is able to provide flexible fitting for arbitrarily complex relationships by the universal approximation theorem [22]. An MDN is therefore defined as a mixture model, where the mixture components are modelled using a neural network.

**Fig 1:**
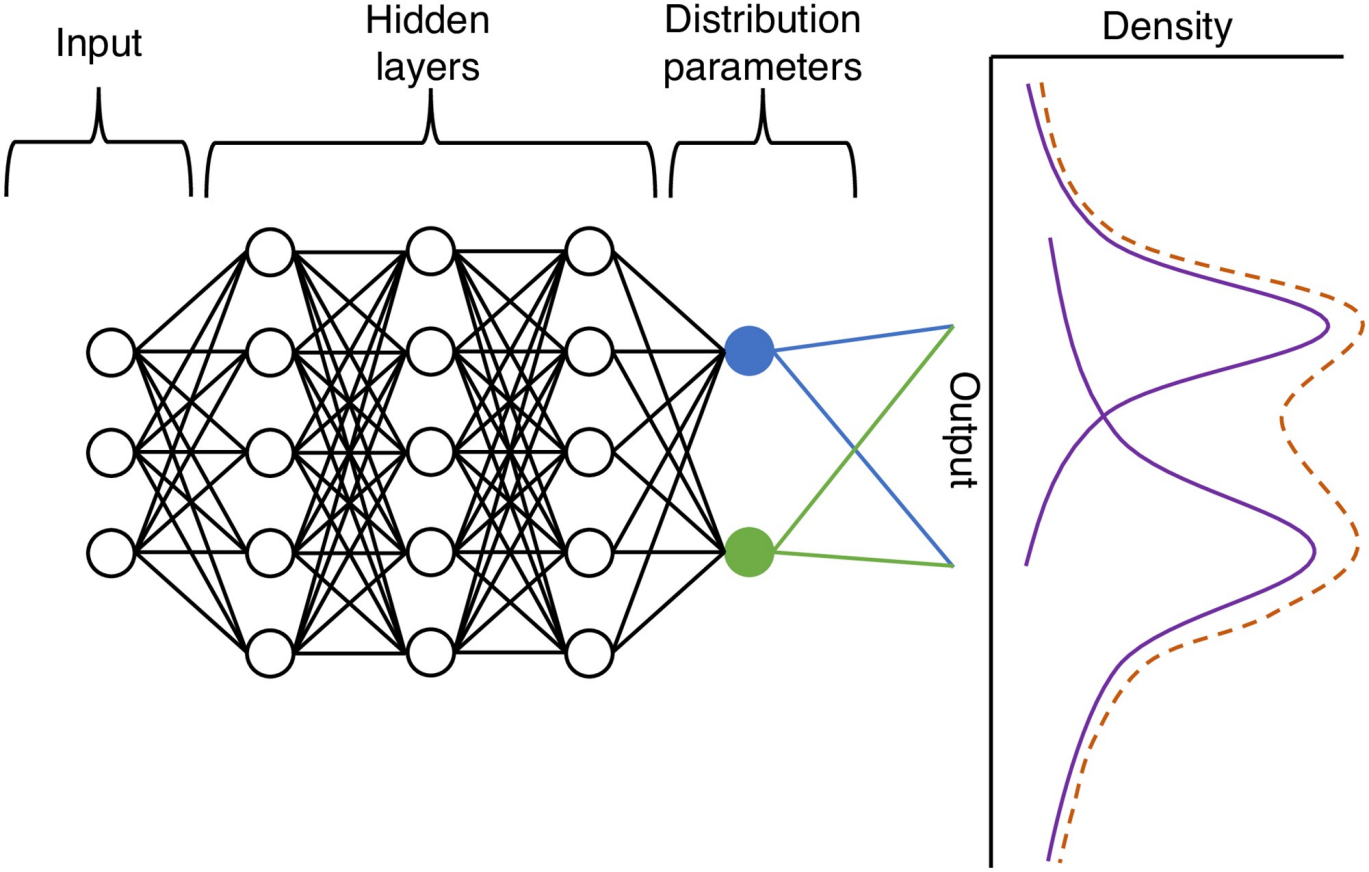
MDN that emulates a model with three inputs and a one-dimensional output with two mixtures. The inputs are passed through two hidden layers, which are then passed on to the normalised neurons, which represent the parameters of a distribution and its weights e.g. the mean (shown in blue) and variance (shown in green) of a normal distribution. These parameters are used to construct a mixture of distributions (represented as a dashed line).

Fig 1 provides an overview of the MDN construction. The inputs of the model *α* are initially fed into the MDN (three such inputs in the example diagram). These are then passed through a number of hidden layers in the neural network, which provide a compact representation of the relationship between the inputs and the unnormalised inputs into the mixture model. These distribution parameters are then passed through a normalisation layer; the weights of the mixture are transformed such that they sum to one and the shape parameters are transformed so that they are positive. These parameters are used to construct the mixture model, where one can draw samples or calculate statistics, such as mean and variance, for a given input. For multiple outputs the final layer can be copied with independent parameters for the number of outputs being considered. Note that a number of aspects of the MDN need to be specified including the number of input parameters, the dimension of the output, the distributions used in the mixture density, and the number and size of the hidden layers.

The MDN can then be fit to the following objective loss function, which is equivalent to maximising the likelihood given in Eq (5),

$$\begin{array}{c} f\left(x|\Omega ,\Theta \right)=-\sum_{i=0}^{n-1}\log(\sum_{j=0}^{m-1}\omega_{j}p_{j}\left(x_{i}|\theta_{j}\right)). \end{array}$$ (6)

Note that provided *p*_*j*_ is differentiable with respect to *θ*_*j*_, this loss represents a differentiable function. Standard techniques based on stochastic gradient descent can then be applied in order to optimise the weights of the network with respect to this loss [23].

### Performance on a simple model

In order to examine how a fitted MDN can capture the broad statistical properties of a distribution, where the underlying mixture distributions differ significantly from the true distribution, we explored fitting to a negative binomial model. The negative binomial can be parameterised by a mean *m* and a shape parameter *k* using the following probability mass function,

$$\begin{array}{c} f\left(x|m,k\right)=\frac{\Gamma (x+k)}{x!\Gamma (k)}{\left(k/\left(m+k\right)\right)}^{k}{\left(m/\left(m+k\right)\right)}^{x}. \end{array}$$ (7)

The parameter *m* defines the mean of the distribution and the shape parameter *k* controls the heterogeneity of the distribution, where the variance is *m*(1 + *m*/*k*). As *k* goes to infinity, the distribution approaches the Poisson distribution.

An MDN was fitted to the negative binomial distribution in the following way. An MDN with 20 gamma mixtures with 3 dense layers of 64 neurons was constructed. Data were sampled as the random output from 1,000 (*m*, *k*) pairs, which were uniformly taken at random from *m* with range 0–100 and from *k* with range 0.01–5. Hence, one data point is a (*m*, *k*) pair and an associated sample output from the negative binomial distribution. The fitting was performed for 150 epochs with a batch size of 50. One epoch is defined as one forward and backward pass through all data points, and the batch size is the number of data points used in a single iteration.

**Fig 2:**
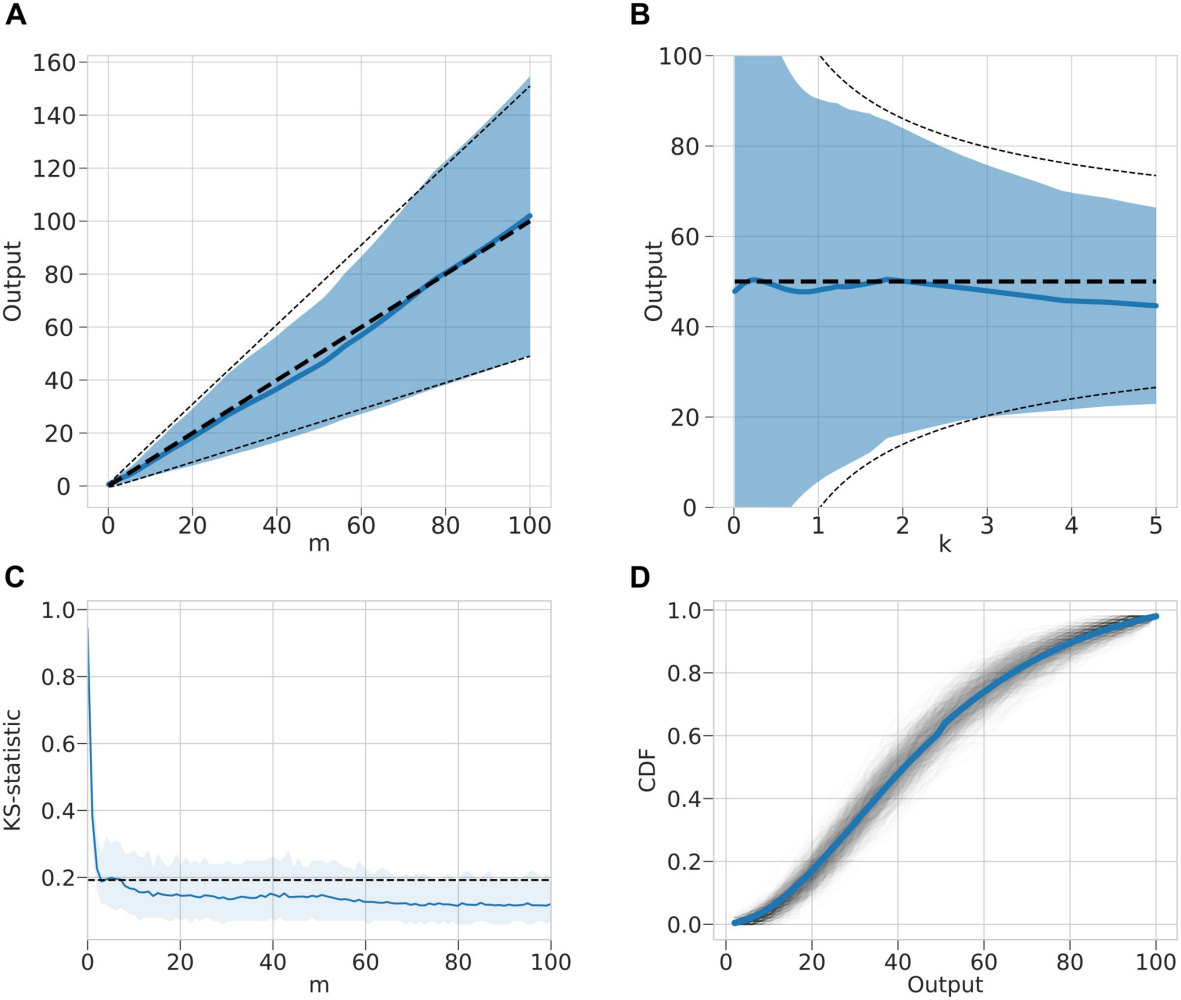
Gamma-MDN output emulating a negative binomial model. (A) For fixed shape parameter *k* = 2.5, the distribution of output from MDN is shown in blue (mean = solid line, variance = shaded region), the theoretical values are shown as a black dashed line (mean = bold line, variance = normal line). (B) For fixed mean parameter *m* = 50, the distribution of output from MDN over a range of *k* values is shown in blue (mean = solid line, variance = shaded region), the theoretical values are shown as a black dashed line (mean = bold line, variance = normal line). (C) Corresponding two-sample K–S statistic where sample of 100 points are drawn from a negative binomial and the MDN over a range of *m* values. 100 replicates are used to estimate a mean K–S statistic and a 95% range. The dashed line represents significance at *α* = 0.05, with values less than this indicating that the two samples do not differ significantly. (D) Example empirical CDFs drawn from 100 samples of MDN with inputs *m* = 50 and *k* = 2.5. 1,000 empirical CDFs are shown as black transparent lines and true CDF is shown as a blue solid line.

In order to compare the statistical properties between the true negative binomial distribution and the MDN emulator a number of tests were devised. First the mean and variance of each distribution were compared by fixing one of the parameters to the mid-point of the parameter range and varying the other parameter (Fig 2A and 2B). In order to statistically compare between a sample generated from the true process and generated from an emulator, the two-sided Kolmogorov–Smirnov (K–S) test was performed on two samples of size 100 across a range of input values for 100 replicates (Fig 2C) [24]. Although, we expect for more complex models the emulated and simulated distributions to exactly match the K–S test provides a quantifiable measure of how close these distributions align and also provides a direct statistic where regions of parameter space give poor emulator performance. The true cumulative density function (CDF) and the empirical CDFs were also compared (Fig 2D).

This experiment broadly captures how well the MDN can emulate a distribution significantly different to its underlying mixture distribution, as well as how capable it is to adequately deal with highly heterogeneous data, which can complicate model fitting [15]. Example code using the accompanying open-source library is given in Box 1.

**Box 1. Code for negative binomial distribution example**

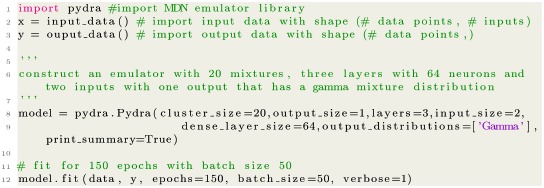

### Stochastic SIR model

The stochastic susceptible–infected–recovered (SIR) model is a standard epidemiological model for the infection dynamics of an epidemic. It is a Markov process where each individual of a population of fixed size *N* can be in any one of the three states: *S*, *I* or *R*. Due to the stochasticity of the model, the output is multi-modal, where identical input parameters can have both quantitatively and qualitatively different outputs, depending on whether an epidemic does or does not occur. The transitions for the process are defined by:

$$\begin{array}{c} (S,I,R)\to (S-1,I+1,R)\;\text{at}\;\text{rate}\;\beta SI/N, \end{array}$$

$$\begin{array}{c} (S,I,R)\to (S,I-1,R+1)\text{at}\;\text{rate}\;\gamma I. \end{array}$$

We generate multiple realisations of the stochastic SIR model and use this data to fit a series of MDNs such that we can evaluate the performance of the emulation model. We note that while there are already fast methods to draw realisations of the process (Gillespie, tau-leap method), we assess this method to understand the benefits for when we want to emulate data from a more computational intensive model [25, 26]. Here, we consider training three different MDNs for different inputs and output distributions.

Firstly, we consider the final size distribution of the epidemic model, which is given as the total number of individuals that have been infected (calculated as *N*(∞) − *S*(∞)). For our data to train an MDN, we take 10,000 realisations of the simulated process using model parameters *β*, sampled from *U*(0, 1), and *γ*, sampled from *U*(0.1, 1), with a population size of *N* = 1, 000, which each give us a final size. To fit an MDN for these two inputs (*β* and *γ*) and one output (final size), we choose a mixture of 20 binomial distributions, where the binomial parameter *n* = 1, 000 is fixed and *p* is learnt from the MDN. We use binomial distributions as the final size is integer valued with a maximum value of the population size. We train on a network with 3 dense layers of 64 neurons for 150 epochs with a batch size of 50. From the trained MDN, new inputs for *β* and *γ*, give us a distribution of the final size.

**Fig 3:**
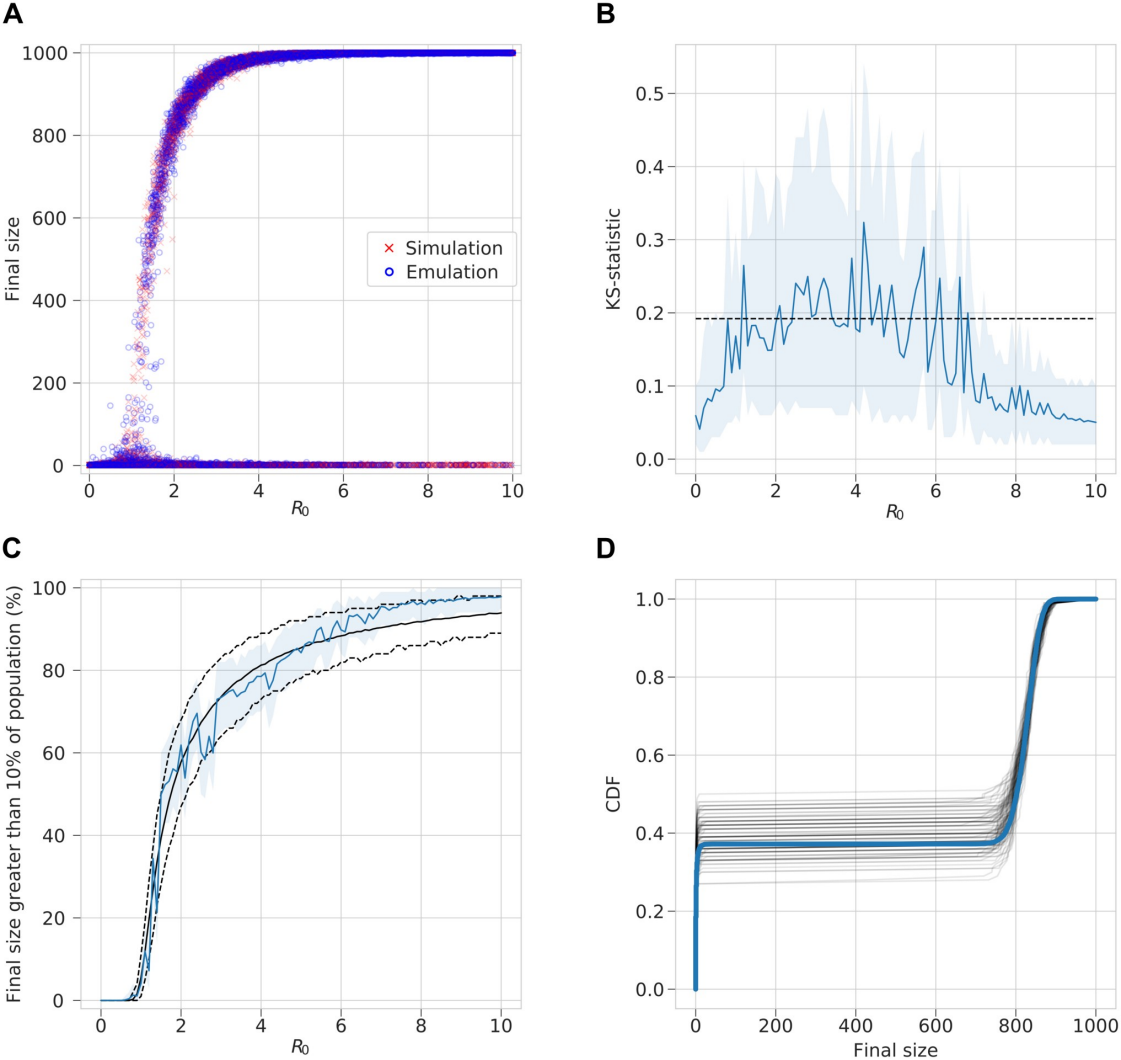
Binomial-MDN output emulating the final size distribution of a stochastic SIR model. (A) For random uniform sampling over *β* and *γ* a sample of the output from MDN across values for the basic reproductive number *R*_0_ = *β*/*γ* are shown in blue and the directly simulated values are shown in red. (B) Corresponding two-sample K–S statistic where sample of 100 points are drawn from a negative binomial and the MDN over a range of *R*_0_ values. 100 replicates are used to estimate a mean K–S statistic and a 95% range. Dashed line represent significance at *α* = 0.05, with values less indicating the two samples do not differ significantly. (C) The percentage of 1,000 realisations of the stochastic SIR model with final size greater than 100 is shown in black with dashed line showing a 95% range. Emulated results are shown by the blue line with a 95% range. (D) Example empirical CDFs drawn from 100 samples of MDN with inputs *β* = 0.4 and *γ* = 0.2. 1,000 empirical CDF are shown as black transparent lines and true CDF is shown as a blue solid line.

Due to the multi-modality, unlike for the negative binomial distribution, calculating the mean and variance of this whole distribution is not a useful concept and so we consider the similarity between the simulated and emulated distributions, by sampling from both (Fig 3A). To quantify their similarity, the two-sided Kolmogorov–Smirnov test was performed on two samples of size 100 (for simulated and emulated data) for randomly sampled *β* and *γ* values for 100 replicates (Fig 3B). Furthermore, since the distribution is bi-modal, we compare the proportion of realisations where the final size is greater than 10% of the population size, indicating that an epidemic has occurred (Fig 3C) and we also compare the empirical CDFs (Fig 3D). These tests were performed across a range of *R*_0_ = *β*/*γ* values.

**Fig 4:**
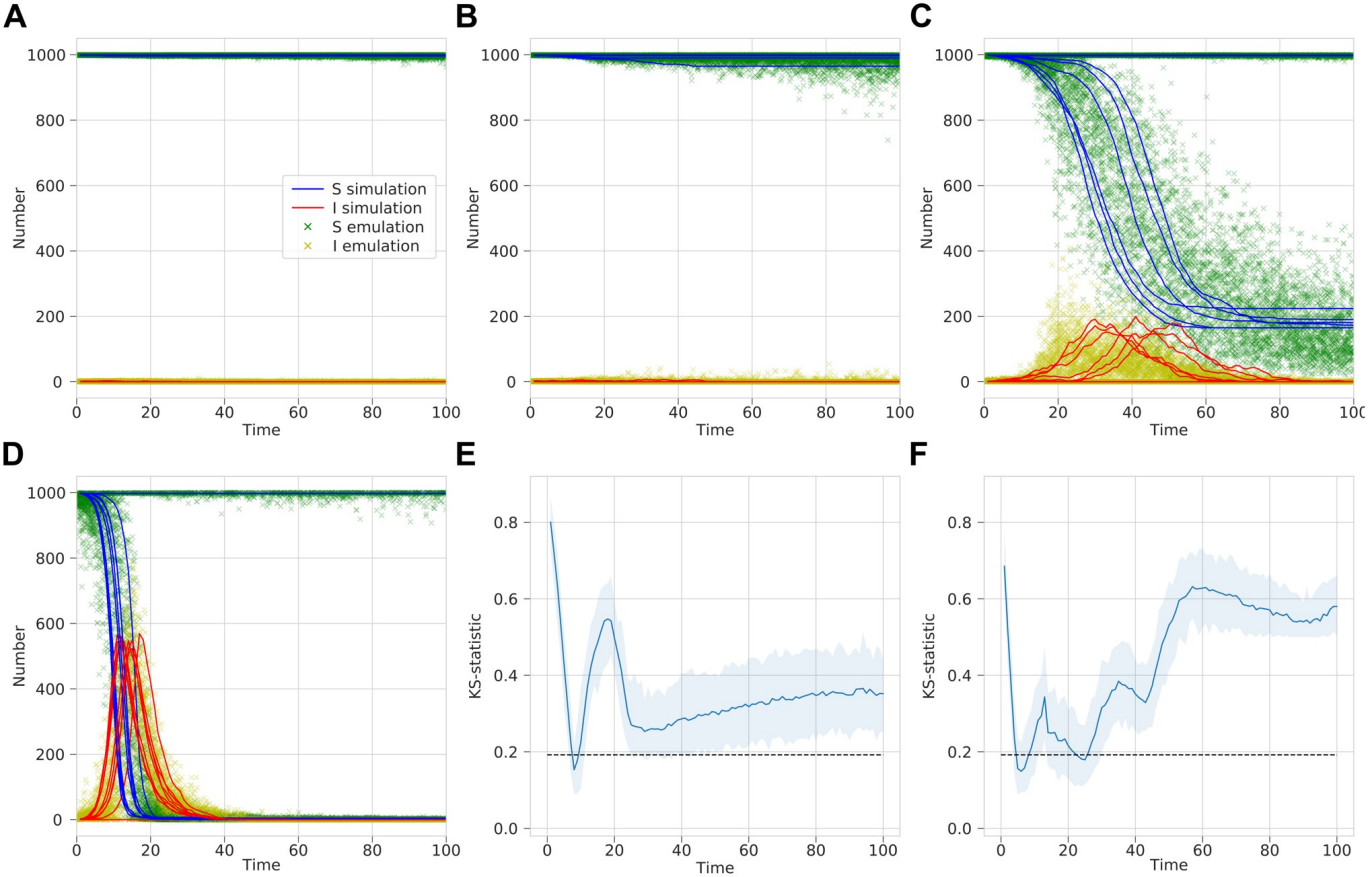
Beta-MDN output emulating the infection dynamics with time for a stochastic SIR model. (A–D) A comparison of simulation results with sampled MDN output for fixed *γ* = 0.2 and *N* = 1, 000 and different *β* values that give the following *R*_0_ values: (A) *R*_0_ = 0.5, (B) *R*_0_ = 1.0, (C) *R*_0_ = 2.0, and (D) *R*_0_ = 5.0. (E–F) Two-sample K–S statistic where sample of 100 points are drawn from a negative binomial and the MDN over a range of time *t* values. 100 replicates are used to estimate a mean K–S statistic and a 95% range. Dashed line represent significance at *α* = 0.05, with values less indicating the two samples do not differ significantly. Tests are for (E) number of susceptible people and (F) number of infected people.

Secondly, we explored the infection dynamics across time. We take 10,000 realisations of the simulated process using model parameters: *β*, sampled from *U*(0, 1); *γ*, sampled from *U*(0.1, 1); and time *t*, sampled as a random integer between 1 and 100. The population size remains fixed with *N* = 1, 000. An MDN was trained on this data with two outputs, the prevalence of susceptible and infected individuals, for the three inputs of *β*, *γ* and time (scaled to be between 0.01 and 1). The prevalence of recovered individuals can be inferred from this given the fixed population size. Since the aim was to learn the prevalence rates, the number in each compartment divided by the population size, we use a mixture of 20 beta distributions, as the beta distribution is defined on the interval [0,1]. The MDN has 3 dense layers of 64 neurons and is trained for 50 epochs with a batch size of 50. For given values of *β*, *γ* and *t*, the trained MDN gives us two output distributions for the number of susceptible and infected people. The simulated and emulated distributions are compared and a two-sample K–S test was performed on the output (Fig 4).

Finally, we added an additional transition for the process to model vaccination of susceptible individuals, which is given by

$$\begin{array}{c} (S,I,R)\to (S-1,I,R+1)\text{at}\;\text{rate}\;\delta S. \end{array}$$

**Fig 5:**
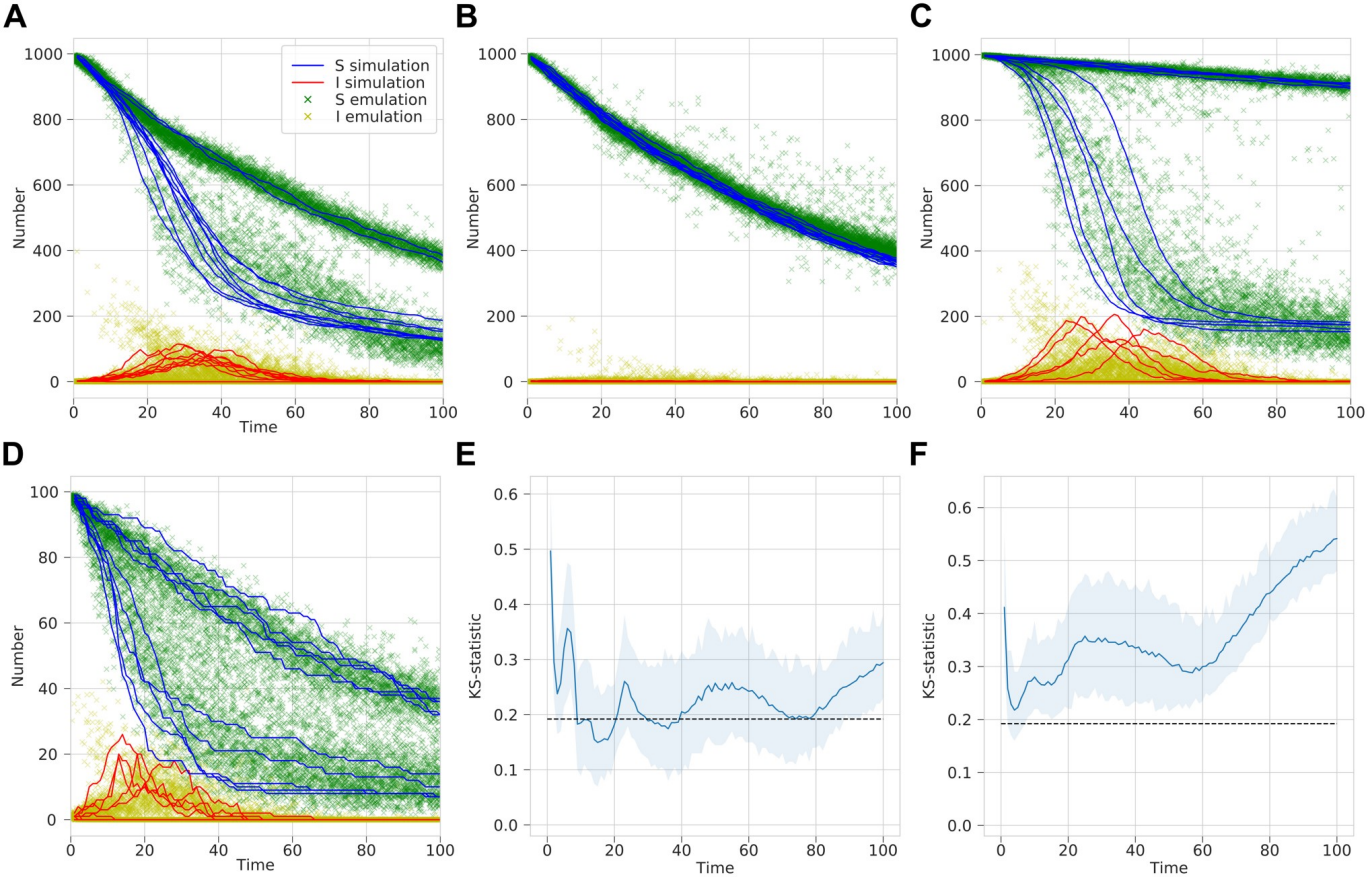
Beta-MDN output emulating the infection dynamics with time for a stochastic SIR model. (A–D) A comparison of simulation results with sampled MDN output for fixed *γ* = 0.2 and different *β*, *δ* and *N* values such that (A) *R*_0_ = 2.0, *δ* = 0.01 and *N* = 1, 000, (B) *R*_0_ = 1.0, *δ* = 0.01 and *N* = 1, 000, (C) *R*_0_ = 2.0, *δ* = 0.001 and *N* = 1, 000, (D) *R*_0_ = 2.0, *δ* = 0.01 and *N* = 100. (E–F) Two-sample K–S statistic where sample of 100 points are drawn from a negative binomial and the MDN over a range of time *t* values. 100 replicates are used to estimate a mean K–S statistic and a 95% range. Dashed line represent significance at *α* = 0.05, with values less indicating the two samples do not differ significantly. Tests are for (E) number of susceptible people and (F) number of infected people.

We then used the same method as before, with the 2 extra inputs of the vaccination rate *δ* sampled from *U*(0, 0.01) and population size sampled as a random integer between 1 and 1,000 (both linearly scaled to have a maximum of 1). Thus, using the trained MDN, given inputs *β*, *γ*, *t*, *δ* and *N*, we output the emulated distribution of the number of susceptible and infected people. Again, the simulation and emulation was compared and a K–S test performed (Fig 5).

## Results

### Simple model

The fitted gamma-MDN emulator was able to broadly capture the mean and variance of the distribution over a range of inputs parameters (Fig 2A and 2B). There were some notable deviations to these statistics however, where the parameters were near the edge of the range. In particular, where *k* < 1, both the mean and variance begin to differ significantly from the true distribution, where the variance increases to infinity as *k* goes to zero (Fig 2B).

The K–S statistic is below the significance level over a broad range of *m* values indicating a sample drawn from the true process and the emulator are similar (Fig 2C). Only when *m* is close to zero, at the edge of the range of training data, does the distribution differ significantly from the true distribution according to the K–S statistic. This can also be broadly shown by plotting example empirical CDF against the true CDF (Fig 2D).

### Stochastic SIR model

The three MDNs were capable of emulating the behaviour of the stochastic SIR model. When sampling from the output distribution of the final size of an epidemic, there was good agreement with the results of sampling from the actual simulation, which is true across the full range of *R*_0_ values (Fig 3A). This is corroborated by the results of the K–S test, where the K–S statistic lies below or close to the significance level defining whether the samples could be drawn from the same distribution, for different *R*_0_ values (Fig 3B). We note that since the distributions are, of course, not exactly the same, drawing more samples would give a weaker result. However, the result was stronger for both small and large *R*_0_ values, with some divergence for the intermediate values, where *R*_0_ is close to 1, where the final size takes a larger range of values. Comparison of the proportion of samples that exhibit stochastic fade-out, as opposed to the emergence of an epidemic, was determined where the final size reaches at least 10% of the population (Fig 3C). These proportions matched closely, with the emulation only slightly deviating to a higher than expected proportion for large *R*_0_ values. Relatively few samples were close to 10% of the population except where *R*_0_ was close to 1, where the proportion would be more sensitive to the threshold definition. The results also matched for the CDFs of the two distributions, shown for fixed *R*_0_ = 2 with *β* = 0.4, *γ* = 0.2 (Fig 3D).

The addition of emulating the actual infection dynamics against time shows that a single trained MDN captures the complexity of varying *R*_0_ by qualitatively reproducing when an epidemic occurs in the correct timescales (Fig 4A–4D). However, statistically comparing the two output distributions for both susceptible and infected individuals, the K–S statistic increases above the significance level (Fig 4E and 4F). This is in part as the beta distribution is a continuous distribution, whereas the simulated prevalence values are all fractions of the population size *N* = 1, 000. The effect of this is particularly stark for small time *t* values where the number of individuals will always be exactly *S*_0_ = 999 and *I*_0_ = 1; there will be some variation outside these values in the emulation, as it is not integer valued. Rounding the emulated values or choosing an integer-valued output distribution helped to improve the K–S test results (see S1 Appendix).

Furthermore, the emulation coped well with learning to reproduce the distributions with further model parameters, vaccination rate *δ* and population size *N*, since inputting different values for *β*, *γ*, *δ* and *N* into the trained MDN qualitatively reproduces the infection dynamics of the simulation across all time values *t* (Fig 5A–5D). With the added complexity of more input parameters, there are some values where the MDN emulation differed from the simulation however, particularly small times (*t* < 30) in the number of infected. Similarly to the MDN without variable vaccination and population size, the results of the K–S test suffer from a similar problem of comparing discrete and continuous distributions, but the resulting distributions were very close at some values (Fig 5E and 5F).

## Discussion

Model emulation is quickly becoming an important and necessary method within infectious disease epidemiology due to the increased use of complex, computationally-intensive models, increased use of direct data fitting requiring many model queries, and increased demand for models to be made directly available to knowledge users [27]. We have explored the use of mixture density networks (MDNs) in order to provide a scalable, flexible solution to this type of emulation [28]. These are mixture models where the underlying parameters of the mixture are neural networks. This allows the significant progress in neural networks and deep learning to be incorporated into the emulation. As neural networks allow for flexible memorisation and interpolation, they provide a compact statistical representation of complex data allowing for rapid inferences to be made.

The main alternative to MDNs for the emulation of a stochastic model are Gaussian processes [29]. These represent the outputs of a model as a multivariate normal distribution. This allows for the quantification of uncertainty and for covariance between points in the model’s input space. While the typical underlying assumption that the data is normal can make GPs restrictive as to the type of data they can represent, they can instead be used to mimic hyperparameters of the output distribution, rather than the output itself [30], allowing more flexibility. We have demonstrated that an MDN can be applied to both overly-dispersed count data (negative binomial example), as well as bimodal count data with a finite domain (final size distribution example). These examples would be inappropriate to apply a GP to, although a viable solution could be to introduce a mixture distribution with parameters described with GPs, rather than the neural network.

Our presented examples expose the potential for MDNs to be used more extensively for model emulation and have scope to be extended in many different ways. For example, in emulating the infection dynamics in Fig 5, we use the input parameters of *β*, *γ*, *δ*, *N*, and *t* to output values of the number of susceptible and infected people. This means that we use the emulator to predict individual time points when sampled from, rather than a whole time series. A possible limitation of this approach is that the emulator may not correctly estimate where the initial epidemic occurs (see Fig 5). Despite this, there would be scope to train an emulator with the further inputs of the number of susceptible and infected people at the previous time point. This MDN could then be sampled from the model iteratively to produce a whole time series. An additional potential issue is that the presented models do not conserve total population size, with it possible for the emulated output to give a total number of people larger than the population size (although there are bounds within each class being larger than the population size). There could be several solutions if this was considered a problem. For example, the output could instead be the sum of infected and susceptible people, along with the proportion of these people in the infected class, constrained to be between zero and one.

The use of an MDN emulator for a stochastic model are two-fold. As the neural network directly learns parameters of the mixture distributions, these may be used directly in the output by, for example, estimating the mean and variance at each point. The emulator may also use the learned distributions to perform random draws from the emulator representing a realisation of the stochastic process. As the emulator approximates the distribution of the output given model input, this essentially produces a likelihood of the data point given the model parameters. Such a synthetic likelihood could then conceivably be used in a Bayesian inference scheme, such as in a approximate Bayesian computation [15]. It would be interesting to apply this approach where a model likelihood is computationally intractable.

We also note, the training of neural networks can lead to the vanishing/exploding gradient problem [31]. Techniques such as momentum and improved initialisation can help mitigate these issues [32]. Anecdotally, we found that re-initialisation with a smaller learning rate generally resolved issues encountered in training. There are also no clear rules on how many epochs training should last or what batch size to use and so the implementer needs to experiment with these parameters to achieve a good fit. In addition, training of neural networks typically involve a large amount of data. If these can be readily generated from a model then an MDN provides a feasible approach to emulation. However when data is small, either a GP or a simplified neural network based on model summary statistics may be a more appropriate approach.

When model computation is slow or there is a large number of input parameters, a more efficient sampling scheme of the parameter space may be appropriate [33]. Efficient high-dimensional sampling schemes such as entropy maximisation have been implemented previously in GPs [14, 34]. As these techniques also involve an approximated likelihood of the data, they could be readily implemented into an MDN scheme, where learning can be conducted in an online fashion.

It is also important to consider the types of appropriate distributions to emulate the model output. Whether they are discrete (e.g. binomial or Poisson) or have finite support (e.g. binomial) can impact the resulting approximation. For example, using a mixture of normal distributions to describe a finite population would lead to some probability of the population being negative. When the population is small this would be non-negligible (see S1 Appendix). It is therefore important to understand the nature of the data being approximated, for example a final size distribution can be well approximated by a Poisson distribution under certain conditions [35]. Plotting the emulated and real data, either as their summary statistics or as a point cloud, as was done here, is an important step toward understanding the validity of the emulator approximation. We found that regular checking of these statistics was necessary to ensure the emulator had converged. We also employed the K–S statistic as a measure of similarity between emulated and real data. A limitation with this statistic is as a mixture distribution is only an approximation to the true distribution, we would expect to see worse performance as the number of samples increase. The K–S statistic can therefore be applied to give a relative measure of emulator performance and highlight regions of parameter space that may exhibit a worse approximation.

Neural networks and in particular deep learning has made enormous progress recently, rapidly improving the state-of-the-art in representation of data sets [36]. This has also led to an increase in open software for developing neural networks including Keras and Tensorflow [37, 38]. Building from these allow the rapid development of new emulation models and provide the use of established code for the testing and analysis of the trained models. In companion to this article, we provide an open access Python library to develop an MDN emulator with example notebooks demonstrating its use. The library also provides details on the exportation of a trained emulator into a web application. For more information, see the accompanying Python library: https://github.com/QCaudron/pydra.

### Conclusion

Mixture density networks have the potential to be used as emulators for complex epidemiological agent-based and micro-simulation models. These techniques incorporate cutting-edge advances in machine learning that provide the possibility to leverage new software libraries in order to perform fast emulator fitting. Applications can include the building of web interfaces for models as well as in model fitting. We hope this technique will prove useful to the broad epidemiology modelling community and as such have included an accompanying open-source library with examples demonstrating its use.

## Supporting information

S1 Appendix Details on the choice of distribution in mixture density network. (PDF)
