# Supplementary material for: The use of mixture density networks in the emulation of complex epidemiological individual-based models
Source: PLoS Comput Biol. 2020 Mar 16;16(3):e1006869. doi: 10.1371/journal.pcbi.1006869 (PMC7098654; doi:10.1371/journal.pcbi.1006869)
Supplement: S1 Appendix — (PDF) [file pcbi.1006869.s001.pdf]

S1 Appendix
The use of mixture density networks in the emulation of complex epidemiological individual-based model

Christopher N. Davis[1,2], T. Deirdre Hollingsworth[3], Quentin Caudron[4], Michael A. Irvine[4,5*]

* Corresponding author: m.irvine@math.ubc.ca
1 MathSys CDT, Mathematics Institute, University of Warwick, Coventry, CV4 7AL, UK
2 Zeeman Institute (SBIDER), University of Warwick, Coventry, CV4 7AL, UK
3 Big Data Institute, Li Ka Shing Centre for Health Information and Discovery, Nuffield Department of Medicine, University of Oxford, Oxford, United Kingdom
4 Scai analytics Ltd., Vancouver, Canada
5 nstitute of Applied Mathematics, University of British Columbia, Vancouver, Canada

The mixture density network (MDN) can be fitted using different types of distribution and the choice of distribution is essential in achieving a mixture distribution that matches well with the empirical distribution of the original inputs. In choosing an appropriate distribution, the support of the distribution should be considered such that it matches the original output being used to train the network.

For the final size distribution results (Fig 3), the final size is given by the total number of individuals infected over the course of the epidemic (calculated as $N(\infty) - S(\infty)$). Since, the population size used is $N = 1,000$ and the initial conditions are for 1 infected person with 999 susceptible, the final size can then take an integer value between 1 and 1,000 inclusive.

We test the results for five different distributions: normal, gamma, beta, Poisson and binomial. The training data was 10,000 samples of the actual simulated process using input parameters β and γ, as described in the main manuscript. For the continuous distributions (normal, gamma and beta), in the training process the output final size of the simulation was linearly scaled to be in the interval $[0.000001, 0.999999]$, such that the beta distribution, which has finite support of the interval $(0, 1)$, could replicate these results in the emulation. For the discrete integer distributions (Poisson and binomial), one was subtracted from the simulated output, such that the output training data was between 0 and 999; this means that a final size of 0 cannot be given by the emulated distributions when the data is scaled back to be between 1 and 1,000.

For the normal distribution, the emulation has a poor fit, since the re-scaled output can be below 1 or more than 1,000 (Fig S1A). This is exemplified in the two-sample Kolmogorov–Smirnov (K–S) test (Fig S1B), where the K–S statistic is much larger than the threshold for the simulated and emulated distributions to be accepted as being the same distribution, for all R_0 values. Additionally, sampling from the emulated distribution and then rounding the results does not significantly improve the quality of the K–S test (Fig S1C).

The gamma distribution shows an improved fit on the normal distribution (Fig S1D), but since the upper limit is unbounded, the fit in this region remains poor. The model fit is improved further by using the beta distribution, which is bounded at both limits (Fig S1G). K–S tests show that neither distribution are sufficiently similar to the simulated distribution (Fig S1E and H). However, by rounding after sampling from these distributions and re-performing the K–S test the results appear

to be significantly better (Fig S1F and I). This is because a large proportion of the simulated results will be 1 or 1,000, where either the epidemic has died out through stochastic fade-out or the whole population becomes infected; rounding the emulated results achieves this exactly. This effect means the K–S statistic becomes small for small R_0 for the gamma distribution and small for both small and large R_0 for the beta distribution, which is bounded at both limits.

The Poisson distribution, being a discrete distribution, achieves a good fit when the final size is small, but for larger final sizes, since it is unbounded and only specified by one parameter, the fit is much worse (Fig S1J). This is reflected in the K–S test statistic (Fig S1K).

The binomial distribution provides the closest match to the simulated data since it is both integer-valued and bounded at both limits, like the simulated results themselves (Fig S1L). Indeed, the K–S test shows the threshold is met for the simulated and emulated distributions to have come from the same distribution across much of the R_0 value range. Hence, this distribution was used in the main manuscript for the final size distribution.

These results are analogous for other simulated training data; to get the best match in the emulation, the output distribution should be chosen to match the characteristics of the simulated output.

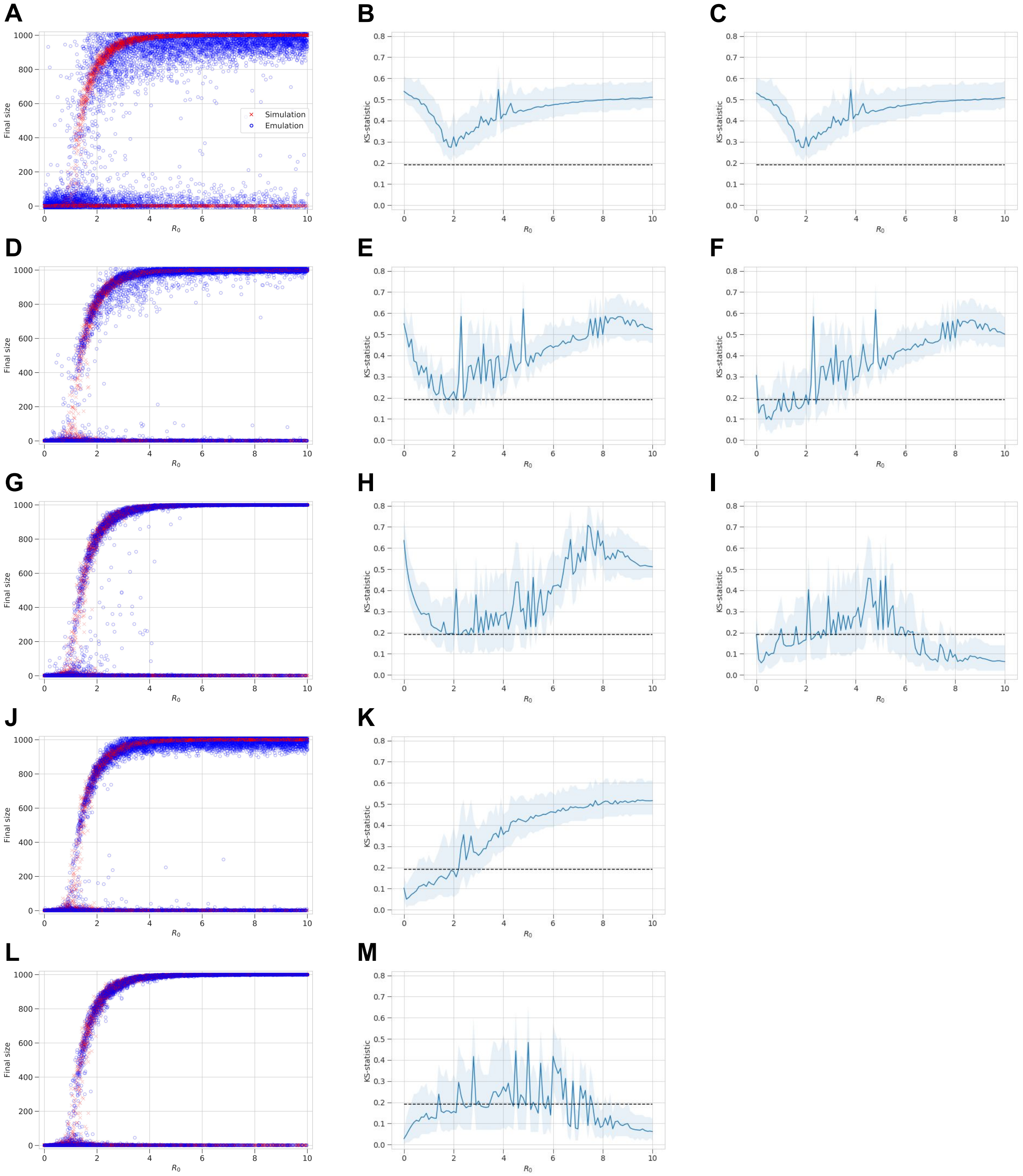

Figure S1: **Emulation and simulation comparison for output MDN distributions**. (A–C) Normal distribution. (D–F) Gamma distribution. (G–I) Beta distribution. (J–K) Poisson distribution. (L–M) Binomial distribution. The first column compares the simulated and emulated output empirical distributions, the second column is the results of a two-sample Kolmogorov–Smirnov test of the simulated and emulated distributions, and the third column repeats the K–S test where the emulated output has been rounded if the chosen output distribution is continuous.
